# Sequential Mediation Effects of First Feeding Practices and Early Breastfeeding Initiation on the Relationship between Prenatal Exclusive Breastfeeding (EBF) Intentions and EBF at 5 Months: A Prospective Special Supplemental Nutrition Program for Women, Infants, and Children Program Infant and Toddler Feeding Practices Study-2

**DOI:** 10.1016/j.cdnut.2026.109557

**Published:** 2026-10-17

**Authors:** Edirisa J Nsubuga, Joel J Komakech, Rahel Mathews, Meng Yang, Tung-Lung Wu, Amanda Conrad

**Affiliations:** 1 Department of Biochemistry, Nutrition, and Health Promotion, Mississippi State University, Starkville, MS, United States; 2 Department of Family and Consumer Sciences, NC Agricultural and Technical State University, Greensboro, NC, United States; 3 Department of Mathematics and Statistics, Mississippi State University, Starkville, MS, United States

**Keywords:** sequential mediation, exclusive breastfeeding, exclusive breastfeeding intentions, first feeding practices, early breastfeeding initiation, WIC, United States

## Abstract

**Background:**

Prenatal exclusive breastfeeding (EBF) intentions are consistently associated with EBF, the best and recommended feeding practice for infants aged <6 mo, yet suboptimal EBF rates remain. Further, evidence on the behavioral pathways underlying this association remains limited, which is crucial for designing and implementing effective child nutrition programs.

**Objectives:**

This study examined the sequential mediation effects of first feeding practices and early breastfeeding initiation on the relationship between prenatal EBF intentions and EBF.

**Methods:**

A prospective Special Supplemental Nutrition Program for Women, Infants, and Children Infant and Toddler Feeding Practices Study cohort of 4367 infant–caregiver dyads was analyzed using sequential mediation analysis. Prenatal EBF intention was measured using the Infant Feeding Intention Scale. First feeding practices were assessed using the very first food/drink the infant was fed after birth. Early breastfeeding initiation and EBF were defined as when the infant started breastfeeding in the first hour after birth, and fed only breastmilk from birth to 5 mo, respectively. Adjusted path coefficients (*β*) of first feeding practices and early breastfeeding initiation on the relationship between prenatal EBF intentions and EBF were reported based on an analytical sample of 2617 observations with complete EBF data. Robustness was assessed through sensitivity and subgroup analyses.

**Results:**

Among 2617 mothers, 48.0% were aged ≥26 y, 61.2% were White, and 22.8% were African American. The sequential indirect effect of prenatal EBF intention on EBF via first feeding practices and early breastfeeding initiation was significant (*β* = −0.062, *P* = 0.009). Indirect effects were observed through first feeding practices (*β* = 0.252, *P* < 0.001) and early breastfeeding initiation (*β* = 0.036, *P* = 0.017), with a significant total indirect effect (*β* = 0.226, *P* < 0.001). The direct (*β* = 0.292, *P* < 0.001) and total effects (*β* = 0.518, *P* < 0.001) were significant, indicating partial mediation. Sensitivity and subgroup analyses yielded consistent findings with the primary model.

**Conclusions:**

Incorporating strategies that promote prenatal EBF intention, early breastfeeding initiation, and discourage prelacteal feeding into child nutrition programs could improve EBF.

## Introduction

The Dietary Guidelines for Americans (DGA) [1], American Academy for Pediatrics (AAP) [2], the United Nations WHO, and the UNICEF [3] recommend that all children should be exclusively breastfed for the first 6 mo to achieve optimal growth, development, health, and survival. Exclusive breastfeeding (EBF) is feeding an infant only breast milk for the first 6 mo, with no other foods or liquids, not even water, except prescribed medicines and vitamin or mineral supplements [4]. The DGA, AAP, WHO, and UNICEF recommend that children initiate breastfeeding within the first hour of birth and be exclusively breastfed on demand, as often as the child wants during day and night, without the use of bottles, teats, or pacifiers [1], [2], [4], [5], [6]. They also recommend introducing safe and adequate complementary foods at 6 mo while continuing breastfeeding ≤2 y or beyond [1], [2], [4], [5], [6].

Breast milk remains the ideal and most nutritious food for infants, especially during the first 6 mo of life [3], [5], [7], [8]. A mother’s breast milk changes in composition and quantity to meet the infant’s changing nutritional needs as the infant grows [3], [5], [7], [8]. Amidst these physiological adaptations, breast milk continues to be safe and clean, and provides antibodies from the mother to the infant. These antibodies develop the infant’s immune system, protecting them against short- and long-term diseases including but not limited to gastrointestinal infections, sudden infant death syndrome, pneumonia, asthma, ear infections, overweight, obesity, and type 1 diabetes [3], [5], [7], [8]. Exclusively breastfed infants perform better on intelligence tests, have higher school attendance, and higher income in adulthood [8]. Breastfeeding improves child development and provides a readily available source of infant food without the need to prepare formula or bottles [5]. Additionally, breastfeeding in general is beneficial to the mother as a birth control method known as lactation amenorrhea and may also reduce the mother's risk of breast and ovarian cancer, type 2 diabetes, and high blood pressure [8], [9], [10]. It also reduces formula food and health costs, leading to economic gains for individual families and the nation at large [11].

Globally, the prevalence of EBF among infants under 6 mo was 47% in 2025 [12]. In the United States, 29.8% of infants are exclusively breastfed from birth to 6 mo [13], which falls short of the 2025 World Health Assembly new global target of 60% by 2030 [14] and the U.S. Department of Health and Human Services’ Healthy People 2030 target of 42.4% by 2030 [15]. In the United States, EBF rates were lowest among mothers with household incomes below 200% of the poverty threshold (26.1%) and among those with only a high school education (25.7%) or less (22.6%), all below the national average [13]. In comparison, non-Hispanic White (31.8%) and non-Hispanic Asian (32.2%) mothers have EBF rates above the national average [13].

Previous studies have shown that EBF practices in the United States are influenced by multiple maternal, infant, household, and behavioral factors, including prenatal EBF intentions, early feeding practices/prelacteal feeding, and early breastfeeding initiation [16], [17], [18], [19], [20], [21], [22]. Additional EBF influences include maternal sociodemographic characteristics, parity, mode of delivery, birth complications, poverty status, food security, and disparities associated with race and ethnicity that may reflect broader social, cultural, and structural influences on breastfeeding practices [16], [17], [18], [19], [20], [21], [22], [23], [24], [25], [26], [27].

Several programs, policies, and initiatives have been implemented in the United States to support mothers in achieving recommended breastfeeding practices [2]. These include healthcare and hospital initiatives [28], [29], [30], [31]; public health and community programs [28], [31], [32], [33], [34], [35], [36]; workplace and legal policies [28], [37], [38], [39], [40]; and education and awareness campaigns [26], [35]. Among these, the Special Supplemental Nutrition Program for Women, Infants, and Children (WIC) program provides eligible breastfeeding mothers with enhanced food packages, breastfeeding support, prenatal breastfeeding toolkits, breast pumps, and access to peer counselors [32], [33], [34].

The decision to exclusively breastfeed is often made prenatally, during pregnancy [41] and prenatal EBF intentions are a strong positive predictor of subsequent EBF practice in the United States [42], [43], [44], [45], [46], [47]. However, the behavioral mechanisms underlying this relationship remain poorly understood, for informing the design and implementation of effective child nutrition programs. Furthermore, prenatal EBF intentions do not always translate into EBF practice [24]. Along the continuum from prenatal EBF intention to EBF practice, several factors, including first feeding practices and early breastfeeding initiation, are associated with EBF outcomes, yet their role within this temporal pathway remains insufficiently understood [48], [49]. This gap underscores the need to adequately assess how prenatal EBF intentions relate to these factors to influence EBF practice for evidence to improve EBF rates in the United States, which have remained suboptimal [13], [31], [50]. Structural equation modeling has provided insights into maternal and child health issues [51], [52]. It offers a useful framework for simultaneously examining complex pathways by evaluating direct and indirect relationships among multiple interconnected variables and assessing potential mediation mechanisms [53], [54]. A sequential mediation approach may therefore provide a more comprehensive understanding of how prenatal EBF intentions translate into EBF practice through early feeding experiences. Therefore, this study examined the sequential mediation effects of first feeding practices and early breastfeeding initiation on the relationship between prenatal EBF intentions and EBF practice at 5 mo. The proposed mediation model is shown in Figure 1.

**FIGURE 1:**
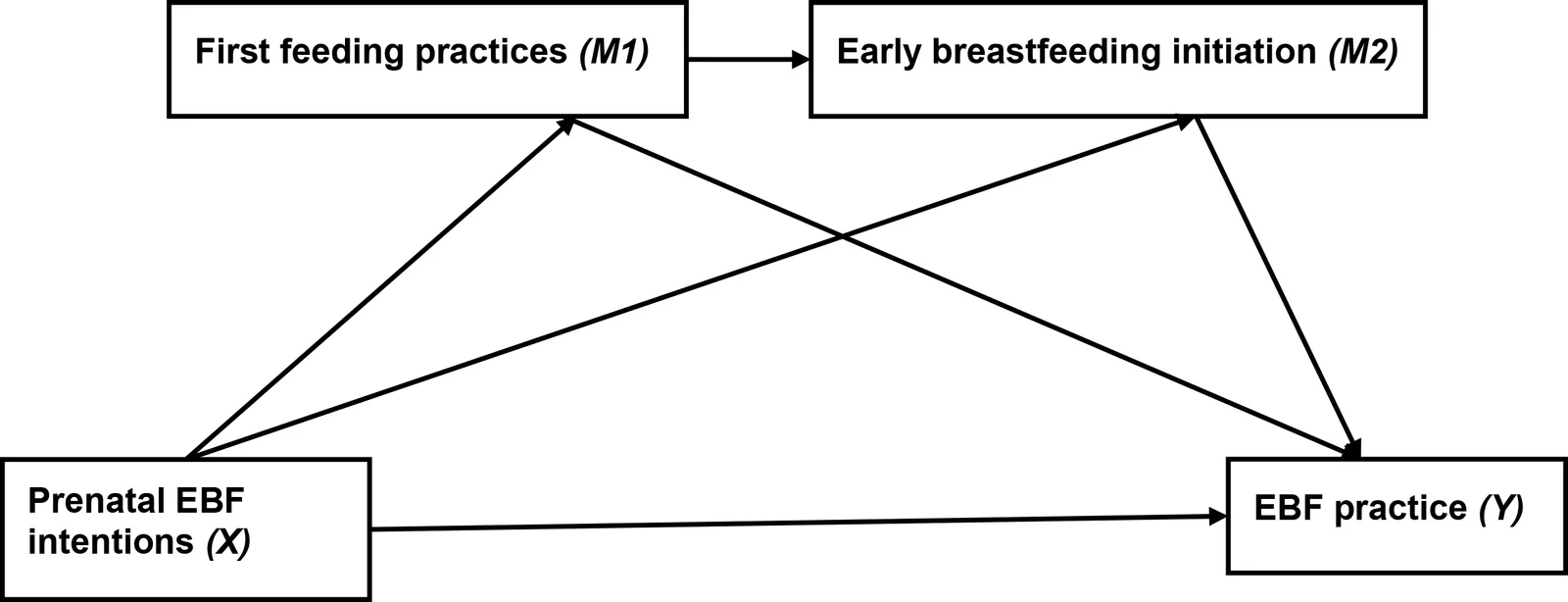
Hypothesized mediation model of first feeding practices and early breastfeeding initiation on the relationship between prenatal Exclusive Breastfeeding (EBF) intentions and EBF practice at 5 months.

## Methods

### Data source, study population, and study design

This study was a secondary analysis of prospective cohort data from the WIC Infant and Toddler Feeding Practices Study (WIC ITFPS-2), commonly known as the “Feeding My Baby Study,” a deidentified, publicly available longitudinal dataset. The WIC ITFPS-2 is a United States national prospective cohort study of 4367 infant–caregiver dyads participating in the WIC program [55], [56]. The WIC ITFPS-2 study recruited a nationally representative sample of 4367 prenatal mothers and mothers of infants aged <6 wk during the summer and fall of 2013 from 80 WIC sites across 27 United States and territories and followed them and their children through 6 y of age, with an additional follow-up survey conducted when the children were aged 9 y [55], [56]. Detailed descriptions of the study design, recruitment procedures, and data collection methods of this dataset have been published elsewhere [55], [56].

### The WIC program

The WIC program is a Federal food and nutrition assistance program administered by the USDA’s Food and Nutrition Service [57], [58]. It was created as a 2-year pilot program in 1972 by an amendment to the Child Nutrition Act of 1966 and was transformed into a permanent program in 1975 [57], [58]. Its mission is to safeguard the health of low-income women, infants, and children aged ≤5 y who are at increased nutritional risk [57], [58]. The WIC program provides nutritious foods to supplement nutrition education, diets, and referrals to health care and other social services [57], [58]. The WIC program serves as a key public health intervention program that goes beyond food assistance by supporting maternal and child health through nutrition education, breastfeeding support, and referrals to health care and other social services [57], [58], [59].

## Measures

### Prenatal EBF intentions

Maternal prenatal EBF intentions were measured during pregnancy using the Der_IFIScale_Cat variable of the ITFPS2 study [60]. This variable reflected categorized EBF intention levels and was presented as an ordinal categorical variable into 5 categories starting from the lowest to the highest EBF intention categories, that is, 0–3.5, 4–7.5, 8–11.5, 12–15.5, and 16, and missing data. These categories were derived from a continuous variable known as the Infant Feeding Intentions Scale (IFIScale). The IFIScale is a validated, 5-item questionnaire developed by Nommsen-Rivers and Dewey [45] to measure a mother’s prenatal intention to initiate and sustain EBF ≤6 mo. It provides a reliable, quantitative prediction of actual breastfeeding duration and exclusivity [43]. The scale scores range from 0 to 16, with 0 indicating no intention to breastfeed and 16 indicating a very strong intention to provide breast milk as the only source of infants’ food for the first 6 mo. Respondents use a 5-point Likert scale (scored from 0 to 4) to answer 5 specific questions. The first 2 questions measure the strength of intention to initiate breastfeeding, that is, I am planning to at least give breastfeeding a try, and I am planning to only formula feed my infant (I will not breastfeed at all), respectively. The last 3 questions assess the strength of intention to provide the infant with human breast milk as the only infant food at 1, 3, and 6 mo of age, respectively. The total score is calculated by averaging the scores of the first 2 questions and summing that average with the scores of the last 3 questions. Detailed information on the scoring of the infant feeding intentions scale is provided in the Supplemental Methods.

### First feeding practices

The first feeding practice was measured using the I1_HF9 variable of the ITFPS2 study [60]. This variable asked about the very first food or drink the infant was fed after birth. It was categorized into formula, mother’s breastmilk, sugar water, plain water, something else, and missing data. In this study, the variable was reclassified into a binary variable with the following categories: mother’s breastmilk and other substances.

### Early breastfeeding initiation

Early breastfeeding initiation was measured using the I1_HF11 variable of the ITFPS2 study [60]. This variable inquired whether the mother started breastfeeding her infant in the first hour after birth or later. It was categorized into first hour, later, and missing data. The variable captured whether breastfeeding was initiated within the recommended first hour after birth or not.

### EBF practice

EBF at 5 mo was measured using the I5_CF1 variable of the ITFPS2 study [60]. The variable reflects current feeding status at the 5-mo interview and is consistent with the current status (24-h recall) method for assessing EBF, a WHO-recognized approach that is currently recommended by WHO and UNICEF for population-level monitoring of EBF [61]. The variable asked whether the mother or anyone else was feeding the child breastmilk either from the breast or from a bottle, formula, or both. It was categorized into only breastmilk, only formula, both breastmilk and formula, and missing data. In this study, the variable was reclassified into a binary variable with the following categories: yes (only breastmilk, indicating EBF) and no (only formula or both breastmilk and formula).

### Covariates

In this study, 7 maternal and household covariates were adjusted for, selected a priori from variables in the dataset [60] based on their theoretical relevance and prior evidence of their associations with the mediators and outcome [16], [17], [18], [19], [20], [21], [22], [23], [24], [25]. These included maternal age at childbirth, maternal education, delivery type, maternal parity, birth complications, household poverty level, and food security during the 6 mo of the child’s life. Maternal age at childbirth was measured by the SL_AgeatBirth_CG variable, which was categorized into 16–19, 20–25, and ≥26 y [60]. Maternal education was measured by the Der_HighestEdu variable, which asked about the mother’s highest education level and was categorized into 9th grade or less, 10th or 11th grade, 12th grade, and more than 12th grade [60]. Delivery type was measured by the SL_DeliveryType variable, which was categorized into vaginal and cesarean [60]. Maternal parity was measured by the SL_Parity variable, which was categorized into first born, second born, and third or subsequent born [60]. Birth complications were measured by the SL_BirthComplication variable, which was categorized into yes and no. Household poverty level was measured by the SL_Poverty2013s variable, which was categorized into 75% of poverty guideline or below, >75% but no more than 130% of poverty guideline, and >130% of poverty guideline [60]. Household food security was measured using the USDA 6-item food security module [62], and the derived variable SL_FoodSecurity was categorized into high or marginal food security, low food security, and very low food security [60].

### Data analysis

The R software version 4.5.1 (R Foundation for Statistical Computing) was used for all the analyses conducted in this study. Descriptive analyses among 2617 participants with complete data on the EBF status were performed to summarize selected background characteristics of the study participants by EBF status at 5 mo (Figure 2). These descriptive characteristics were summarized as frequencies and percentages and compared using chi-square tests because they were categorical. Corresponding *P* values were reported to assess differences between mothers who exclusively breastfed and those who did not. Statistical significance for all the analyses conducted in this study was set at *P* ≤ 0.05. These analyses were conducted to describe the analytic sample and examine differences in participant characteristics by EBF practice at 5 mo.

**FIGURE 2:**
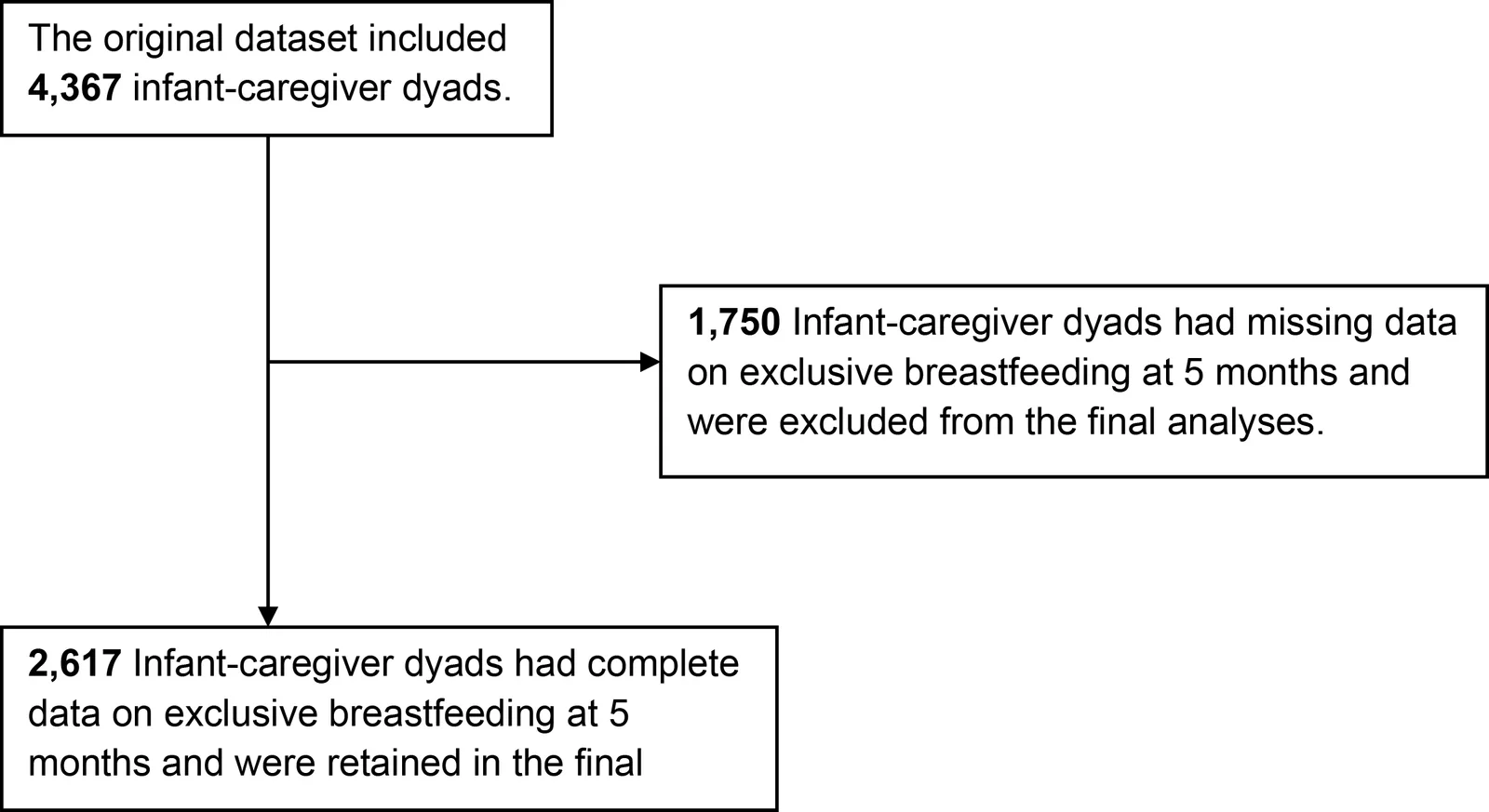
Flow diagram showing the change in the study sample size.

Correlation and cross-tabulation analyses were conducted between first feeding practices and early breastfeeding initiation to assess the degree of association between the 2 mediators and evaluate the potential for multicollinearity within the sequential mediation model [53], [63]. This step was undertaken because highly correlated mediators may introduce estimation challenges, including inflated SEs, unstable parameter estimates, and difficulty distinguishing the unique contribution of each mediator to the indirect pathway [54], [63]. Correlation coefficients (*r*) were interpreted according to established guidelines, with *r* < 0.30 considered weak, *r* = 0.30–0.69 considered moderate and *r* ≥ 0.70 considered strong and indicative of substantial overlap and a potential source of multicollinearity concerns [64], [65]. Cross-tabulations were additionally examined to assess the degree of agreement between the 2 mediators and to determine whether they represented distinct constructs within the hypothesized sequential mediation pathway [53], [66].

A theory-driven sequential mediation model was estimated using structural equation modeling (SEM) [53], [54] to examine the association between prenatal EBF intentions and EBF practice at 5 mo through first feeding practices and early initiation of breastfeeding (Figure 1). First feeding practices were specified as the first mediator and early initiation of breastfeeding as the second mediator in the hypothesized causal pathway. The model included direct and indirect pathways linking the exposure, mediators, and outcome and was adjusted for the 7 covariates identified a priori as potential confounders.

The sequential mediation model was estimated using SEM with the weighted least squares mean and variance adjusted (WLSMV) estimator, implemented in the *lavaan* package in R. Missing data were handled using pairwise present estimation under the WLSMV framework, resulting in a final analytical sample of 2617 observations with complete EBF data (Figure 2). WLSMV was selected because the exposure, mediators, and outcome were ordinal categorical variables and because it provides robust parameter estimates, SEs, and adjusted test statistics without assuming multivariate normality [67], [68]. Because the independent variable and covariates were treated as fixed exogenous variables, observations with missing values on these variables were excluded from the analysis [53], [69]. Statistical inference for direct, indirect, and total effects was based on the robust estimation framework provided by WLSMV, including robust standard errors, Wald *z*-tests, and corresponding 95% Wald-type confidence intervals [53], [69], [70]. Sequential indirect, mediator-specific indirect, total indirect, direct, and total effects were computed as model-defined parameters within the SEM framework and evaluated using robust Wald-based inference rather than bootstrap confidence intervals. Results were reported as standardized path coefficients (*β*), robust SEs, 95% Wald-type confidence intervals, and *P* values [53].

This study employed a theory-driven sequential mediation approach, with covariates and pathways specified a priori based on theoretical and empirical evidence [53], [54]. All prespecified covariates were retained to ensure adequate control of potential confounding [71]. Therefore, the fully adjusted sequential mediation model was retained because it was theoretically justified, provided stronger control of confounding, produced stable estimates, and preserved the hypothesized causal ordering of variables [54]. Model evaluation focused on the estimated direct and indirect effects and their consistency across robustness and sensitivity analyses. These analyses, including alternative model specifications and subgroup analyses, provided a more meaningful assessment of model validity than modifying the model solely to obtain fit statistics [53], [71].

To assess the stability and validity of the sequential mediation findings, 2 sets of robustness analyses were conducted. First, sensitivity analyses were performed by estimating alternative models in which each mediator was included separately. Specifically, models including only first feeding practices and only early breastfeeding initiation were fitted to evaluate the relative contribution of each mediator to the association between prenatal EBF intentions and EBF practice at 5 mo. Second, subgroup analyses were conducted by stratifying the sample according to delivery type (vaginal compared with cesarean section) to assess whether the observed mediation pathways were consistent across delivery contexts. All robustness models were estimated using the WSLMV estimator, which is appropriate for ordinal categorical variables [67], [68], and were adjusted for the same 7 covariates included in the primary analysis. Robustness was assessed by evaluating whether the direction, magnitude, and statistical significance of the estimated pathways remained consistent across alternative model specifications and subgroup analyses.

## Ethical considerations

The Mississippi State University Institutional Review Board (IRB) reviewed the study protocol and determined that it was exempt from the Human Research Protection Program/IRB oversight (IRB-26-002).

## Results

Of the 2617 mothers who had complete data on EBF practice at 5 mo, 48.0% were aged ≥26 y, 61.2% were White, 22.8% were Black or African American, and 15.6% were exclusively breastfeeding at 5 mo (Table 1). There were significant differences in mother’s age at child birth (*P* = 0.014), race (*P* < 0.001), and highest education (*P* < 0.001), delivery type (*P* = 0.002), birth complications (*P* = 0.014), household poverty levels (*P* < 0.001), prenatal EBF intentions (*P* < 0.001), first feeding practices (*P* < 0.001), and early breastfeeding initiation (*P* < 0.001) between the exclusively breastfeeding and not exclusively breastfeeding groups. However, there were no significant differences in maternal parity and household food security between the exclusively breastfeeding and not exclusively breastfeeding groups (Table 1).

**TABLE 1:** Selected background characteristics of the study participants by EBF at 5 mo

Characteristics	Overall (*n* = 2617)		EBF				*P* value
	Frequency (*n*)	Percentage (%)	No		Yes		
			*n*	%	*n*	%	
			2210	84.4	407	15.6	
Mother's age at childbirth (y)							
16–19	297	11.3	263	11.9	34	8.4	0.014^e^
20–25	1064	40.7	911	41.2	153	37.6	
≥26	1256	48.0	1036	46.9	220	54.1	
Mothers race							
Black or African American	597	22.8	547	24.8	50	12.3	<0.001^g^
White	1602	61.2	1319	59.7	283	69.5	
Other	418	16.0	344	15.6	74	18.2	
Mother's highest education^a^							
≤ 9th grade	255	9.8	222	10.1	33	8.2	<0.001^g^
10th–11th grade	382	14.6	345	15.6	37	9.2	
12th grade	977	37.5	844	38.3	133	32.9	
>12th grade	995	38.1	794	36.0	201	49.8	
Delivery type							
Vaginal	1754	67.0	1454	65.8	300	73.7	0.002^f^
Cesarean	863	33.0	756	34.2	107	26.3	
Maternal parity							
First born	1072	41.0	905	41.0	167	41.0	0.448
Second born	721	27.6	600	27.1	121	29.7	
≥Third born	824	31.4	705	31.9	119	29.2	
Birth complications							
Yes	299	11.4	267	12.1	32	7.9	0.014^e^
No	2318	88.6	1943	87.9	375	92.1	
Poverty level at child birth							
≤75% of guidelines	1634	62.5	1423	64.4	211	51.8	<0.001^g^
>75% but ≤130% guidelines	715	27.3	581	26.3	134	32.9	
>130% of guidelines	268	10.2	206	9.3	62	15.2	
Household food security							
High or marginal	1361	52.0	1145	51.8	216	53.1	0.853
Low	815	31.1	693	31.4	122	30.0	
Very low	441	16.9	372	16.8	69	17.0	
Prenatal EBF intentions^b^							
0–3.5 (very low)	82	4.0	82	4.7	0	0.0	<0.001^g^
4–7.5 (low)	445	21.5	430	24.9	15	4.5	
8–11.5 (moderate)	782	37.9	700	40.5	82	24.4	
12–15.5 (strong)	608	29.4	450	26.0	158	47.0	
16 (very strong)	149	7.2	68	3.9	81	24.1	
First feeding practices^c^							
Other foods	856	36.5	827	42.0	29	7.8	<0.001^g^
Breastmilk	1487	63.5	1142	58.0	345	92.2	
Early breastfeeding initiation^d^							
Later	747	40.2	642	43.2	105	28.2	<0.001^g^
First hour	1112	59.8	845	56.8	267	71.8	

Abbreviation: EBF, exclusive breastfeeding.

a Among 2609 mothers with complete data on both variables.

b Among 2066 mothers with complete data on both variables.

c Among 2343 mothers with complete data on both variables.

d Among 1859 mothers with complete data on both variables.

e *P* < 0.05.

f *P* < 0.01.

g *P* < 0.001.

The correlation between first feeding practices and early breastfeeding initiation was moderate (*r* = 0.482), and cross-tabulation revealed substantial variability across categories indicating that the 2 variables capture distinct aspects of early feeding behavior (Table 2). Therefore, both were retained as sequential mediators in the analysis.

**TABLE 2:** Correlation and cross-tabulation between first feeding practices and early breastfeeding initiation (*n* = 2570^a^, *r* = 0.482)

Characteristics	Early breastfeeding initiation			
	First hour		Later	
	Frequency (*n*)	Percentage (%)	*n*	%
First feeding practices				
Breastmilk	1476	96.5	603	57.9
Others	53	3.5	438	42.1

a 2570 mothers had complete data on the 2 variables.

As depicted by the results of the regression analysis between variables from the adjusted primary sequential mediation model, the results indicated significant direct effects (Table 3). Specifically, there was a significant direct effect of prenatal EBF intentions on first feeding practices (*β* = 0.450, *P* < 0.001), early breastfeeding initiation (*β* = −0.170, *P* < 0.001), and EBF practice at 5 mo (*β* = 0.292, *P* < 0.001). Similarly, first feeding practices had a significant direct effect on early breastfeeding initiation (*β* = 0.652, *P* < 0.001), and prenatal EBF practice at 5 mo (*β* = 0.560, *P* < 0.001). Early breastfeeding initiation demonstrated a significant direct effect on EBF practice at 5 mo (*β* = −0.212, *P* = 0.008) (Table 3).

**TABLE 3:** Regression analysis results between variables in the adjusted primary sequential mediation model

Pathway		*β*	Robust SE	*Z*	*P* value
Dependent variable	Predictor variable				
First feeding practices	Prenatal EBF intentions	0.450	0.034	17.06	<0.001^c^
	Mother's age at child birth	0.004	0.053	0.125	0.901
	Mother's highest education	0.108	0.034	4.14	<0.001^c^
	Delivery type	−0.165	0.065	−6.70	<0.001^c^
	Poverty level at child birth	0.097	0.050	3.63	<0.001^c^
	Maternal parity	0.048	0.043	1.64	0.100
	Household food security	0.001	0.040	0.025	0.980
	Birth complications	0.248	0.095	10.04	<0.001^c^
Early breastfeeding initiation	First feeding practices	0.652	0.028	22.0	<0.001^c^
	Prenatal EBF intentions	−0.170	0.039	−5.19	<0.001^c^
	Mother's age at child birth	−0.024	0.057	−0.700	0.484
	Mother's highest education	0.098	0.035	3.34	<0.001^c^
	Delivery type	−0.178	0.070	−6.23	<0.001^c^
	Poverty level at child birth	−0.001	0.053	−0.006	0.995
	Maternal parity	0.052	0.048	1.50	0.135
	Household food security	0.013	0.044	0.445	0.656
	Birth complications	0.142	0.102	5.03	<0.001^c^
EBF practice at 5 mo	Early breastfeeding initiation	−0.212	0.083	−2.67	0.008^b^
	First feeding practices	0.560	0.085	6.45	<0.001^c^
	Prenatal EBF intentions	0.292	0.061	6.03	<0.001^c^
	Mother's age at child birth	0.083	0.070	2.07	0.038^a^
	Mother's highest education	−0.009	0.043	−0.26	0.796
	Delivery type	−0.069	0.093	−1.90	0.057
	Poverty level at child birth	0.040	0.058	1.25	0.210
	Maternal parity	−0.001	0.059	−0.018	0.985
	Household food security	−0.018	0.052	−0.556	0.578
	Birth complications	0.005	0.146	0.134	0.894

Abbreviations: *β*, standardized coefficient; EBF, exclusive breastfeeding; *Z*, *Z*-score.

a *P <* 0.05.

b *P <* 0.01.

c *P <* 0.001.

Several mediation effects were observed in the relation between prenatal EBF intentions, first feeding practices, early breastfeeding initiation, and EBF practice at 5 mo (Table 4). The sequential indirect effect of prenatal EBF intentions on EBF practice at 5 mo via first feeding practices and early breastfeeding initiation was significant (*β* = −0.062, *P* = 0.009). Indirect effects were observed through first feeding practices (*β* = 0.252, *P* < 0.001) and early breastfeeding initiation (*β* = 0.036, *P* = 0.017), with a significant total indirect effect (*β* = 0.226, *P* < 0.001). The direct (*β* = 0.292, *P* < 0.001) and total effects (*β* = 0.518, *P* < 0.001) were significant, indicating partial mediation (Table 4).

**TABLE 4:** Mediating effects of first feeding practices and early breastfeeding initiation on the relationship between prenatal EBF intentions and EBF practice at 5 mo

Pathway	Effect (*β*)	SE	95% Wald CI	*P* value
Sequential indirect: prenatal EBF intentions→ first feeding practices→ early breastfeeding initiation→ EBF practice	−0.062	0.030	−0.137, −0.019	0.009^b^
Indirect 1: prenatal EBF intentions→ first feeding practices→ EBF practice	0.252	0.052	0.215, 0.418	<0.001^c^
Indirect 2: prenatal EBF intentions→ early breastfeeding initiation→ EBF practice	0.036	0.019	0.008, 0.082	0.017^a^
Total indirect effect	0.226	0.042	0.201, 0.366	<0.001^c^
Direct effects	0.292	0.061	0.247, 0.486	<0.001^c^
Total effects	0.518	0.045	0.561, 0.740	<0.001^c^

Abbreviations: CI, confidence interval*;* EBF, exclusive breastfeeding.

a *P* < 0.05.

b *P* < 0.01.

c *P* < 0.001.

When early breastfeeding initiation was considered as the sole mediator, the indirect effect was small but statistically significant (*β* = 0.014, *P* < 0.046) (Table 5). In contrast, when first feeding practices were included as the only mediator, the indirect effect was substantially larger (*β* = 0.190, *P* < 0.001). These findings suggest that first feeding practices represent the predominant mediating pathway linking prenatal EBF intentions to EBF practice at 5 mo, whereas early breastfeeding initiation contributes a comparatively smaller indirect effect. The persistence of statistically significant indirect effects across both alternative model specifications supports the robustness of the primary mediation findings. Significant direct effects were also observed in both alternative models, consistent with the partial mediation pattern identified in the primary model.

**TABLE 5:** Robustness checks of the sequential mediation findings using alternative mediator specifications

Model	Mediation effect	*β*	SE	95% Wald CI	*P* value
Primary sequential mediation model	Sequential indirect effect	−0.062	0.030	−0.137, −0.019	0.009^b^
	Indirect effect via first feeding practices	0.252	0.052	0.215, 0.418	<0.001^c^
	Indirect effect via early breastfeeding initiation	0.036	0.019	0.008, 0.082	0.017^a^
First feeding practices only model	Indirect effect	0.190	0.032	0.175, 0.302	<0.001^c^
Early breastfeeding initiation only model	Indirect effect	0.014	0.009	0.001, 0.034	0.046^a^

Abbreviations: *β*, standardized beta coefficient; CI, confidence interval.

a *P* < 0.05.

b *P <* 0.01.

c P *<* 0.001.

Subgroup analyses stratified by delivery type demonstrated patterns that were generally consistent with those observed in the primary sequential mediation model (Table 6). In both vaginal and cesarean delivery subgroups, the indirect effect through first feeding practices remained statistically significant (vaginal: *β* = 0.290, *P* < 0.001; cesarean: *β* = 0.195, *P* = 0.011), as did the total indirect, direct, and total effects. Although the sequential indirect effect and the indirect effect through early breastfeeding initiation alone did not reach statistical significance within either subgroup, the estimated effects remained in the same direction and were of similar magnitude to those observed in the primary model [sequential indirect effect: *β* = −0.050, *P* = 0.052 (marginally nonsignificant) and *β* = −0.089 in the vaginal and cesarean subgroups, respectively, compared with *β* = −0.062 in the primary model]. The attenuation in statistical significance may reflect reduced precision after stratification, particularly in the smaller cesarean delivery subgroup. Overall, the persistence of significant total indirect, direct, and total effects, together with the consistency in the direction and magnitude of effects across delivery subgroups, supports the robustness of the primary mediation findings (Table 6).

**TABLE 6:** Subgroup analysis of sequential mediation effects by delivery type compared with the primary model

Pathway	Primary model		Vaginal delivery subgroup		Cesarean section subgroup	
	*β*	*P* value	*β*	*P* value	*β*	*P* value
Sequential indirect: prenatal EBF intentions→ first feeding practices→ early breastfeeding initiation→ EBF practice at 5 mo	−0.062	0.009^b^	−0.050	0.052	−0.089	0.080
Indirect 1: prenatal EBF intentions→ first feeding practices→ EBF practice at 5 mo	0.252	<0.001^c^	0.290	<0.001^c^	0.195	0.011^a^
Indirect 2: prenatal EBF intentions→ early breastfeeding initiation→ EBF practice at 5 mo	0.036	0.017^a^	0.028	0.075	0.056	0.108
Total indirect effect	0.226	<0.001^c^	0.268	<0.001^c^	0.162	0.008^b^
Direct effects	0.292	<0.001^c^	0.259	<0.001^c^	0.336	<0.001^c^
Total effects	0.518	<0.001^c^	0.527	<0.001^c^	0.498	<0.001^c^

Abbreviations: *β*, standardized coefficient; EBF, exclusive breastfeeding.

a *P* < 0.05.

b *P* < 0.01.

c *P* < 0.001.

## Discussion

This study examined the sequential mediation effects of first feeding practices and early breastfeeding initiation on the relationship between prenatal EBF intentions and EBF practice at 5 mo. Our findings showed that the association between prenatal EBF intentions and EBF practice at 5 mo was partially mediated through individual and sequential pathways involving first feeding practices and early breastfeeding initiation. Specifically, prenatal EBF intentions affected EBF practice through 3 indirect paths: the relationship mediated by early feeding practices, the relationship mediated by early breastfeeding initiation, and the relationship sequentially mediated by first feeding practices and early breastfeeding initiation. In this study, mothers with stronger prenatal EBF intentions were more likely to provide breastmilk as the very first feed the infant was introduced to after birth, and initiated breastfeeding within the recommended first hour after birth [1], [2], [4], [5], [6], which in turn was associated with exclusively breastfeeding their infants, thereby supporting the hypothesized model.

In this study, stronger prenatal EBF intentions were associated with a greater likelihood of EBF practice among low-income mothers enrolled in WIC, consistent with previous research [47]. This finding suggests that prenatal EBF intentions may reflect maternal motivation, preparedness, and commitment to achieving breastfeeding goals [25], [47]. Mothers with stronger prenatal EBF intentions are likely to possess greater knowledge of the benefits of EBF, more positive attitudes toward EBF, stronger perceived social support, and greater confidence in their ability to successfully breastfeed, all of which may facilitate the initiation and continuation of EBF [25]. These findings can further be explained by the Theory of Planned Behavior, which posits that health behaviors are largely influenced by behavioral intentions that arise from attitudes, subjective norms, and perceived behavioral control [72], [73]. Within this framework, mothers who perceived EBF positively, receive support from family members and healthcare providers, and believe they have the capacity to exclusively breastfeed may be more likely to develop strong prenatal EBF intentions that are subsequently translated into breastfeeding behavior [33]. The consistency of our findings with previous studies further reinforces the importance of prenatal EBF intentions as a key determinant of breastfeeding outcomes [33]. Importantly, this study extends the existing literature by demonstrating that the influence of prenatal EBF intentions on EBF practice operates not only directly but also through modifiable postpartum behaviors, including first feeding practices and early breastfeeding initiation. These findings underscore the importance of strengthening EBF intentions during pregnancy as part of comprehensive strategies to improve EBF rates [74].

This study identified first feeding practices as a mediator of the relationship between prenatal EBF intentions and EBF practice among low-income mothers enrolled in the WIC program. Mothers with stronger prenatal EBF intentions were more likely to provide breastmilk as their infant’s first food after birth, which in turn was associated with a higher likelihood of EBF at 5 mo. This finding suggests that the first feeding decision may represent one of the earliest behavioral manifestations of prenatal EBF intentions [72], [73], [75]. Mothers who are strongly committed to EBF before and during pregnancy may be more likely to avoid formula, water, or other breastmilk substitutes after birth and commit to breastfeed from the onset [47], [74]. Such practices may support the establishment of breastfeeding, reinforce maternal confidence, reduce reliance on supplemental feeding, and promote feeding patterns that are conducive to sustained EBF [47], [74]. Our findings are consistent with recommendations from the revised Baby-Friendly Hospital Initiative (BFHI), which emphasizes avoiding nonmedically indicated supplementation, food or fluids other than breastmilk, and supporting mothers in the establishment of breastfeeding [74]. This study’s findings are also aligned with WHO guidance discouraging prelacteal feeding, which may interfere with breastfeeding establishment and reduce the likelihood of EBF [76], [77]. Even though some previous studies have independently linked prenatal EBF intentions and optimal first feeding practices with improved breastfeeding outcomes [33], [43], [47], our findings further demonstrated that first feeding practices constitute an important mechanism through which prenatal EBF intentions influence later EBF behavior. From a public health perspective, interventions should concentrate not only on strengthening prenatal EBF intentions but also on supporting mothers to provide breastmilk as the infant’s first feed, particularly among socioeconomically disadvantaged populations at increased risk of breastfeeding discontinuation [3], [74].

Early breastfeeding initiation also mediated the association between prenatal EBF intentions and EBF practice at 5 mo. Mothers with stronger prenatal EBF intentions were more likely to initiate breastfeeding within the first hour after birth, which subsequently increased the likelihood of maintaining EBF. These findings highlighted the immediate postpartum period as a critical stage during which prenatal EBF intentions are translated into observable breastfeeding behaviors. According to the Health Action Process Approach, intentions must be converted into concrete actions before they can influence long-term behavioral outcomes [75], and timely breastfeeding initiation may represent an important action phase in this process. Mothers with stronger prenatal EBF intentions may be more prepared and motivated to initiate breastfeeding promptly after delivery, thereby facilitating the early establishment of breastfeeding [74]. Early breastfeeding initiation may also promote mother–infant contact, stimulate breastmilk production, enhance maternal confidence, and support effective breastfeeding patterns, all of which are associated with successful EBF [49]. Our findings were consistent with the BFHI and the WHO recommendation that breastfeeding should begin within the first hour after birth because timely initiation is strongly associated with improved EBF rates [74], [78]. Furthermore, our findings were similar to those of previous studies demonstrating positive associations between prenatal EBF intentions, early breastfeeding initiation, and EBF [10], [42], [79]. These findings suggest that strengthening prenatal EBF intentions alone may be insufficient unless mothers are also supported to initiate breastfeeding promptly after delivery. Interventions that facilitate breastfeeding within the first hour after birth may therefore play an important role in increasing EBF rates among vulnerable populations [34], [74].

Beyond the individual mediating effects, our findings demonstrated a significant sequential pathway involving both first feeding practices and early breastfeeding initiation. Mothers with stronger prenatal EBF intentions were more likely to provide breastmilk as their infant’s first food, which was subsequently associated with a greater likelihood of initiating breastfeeding within the first hour after birth and, ultimately, maintaining EBF, which supports our hypothesized model. This finding suggests that the influence of prenatal EBF intentions on later EBF practice may manifest through a progression of interconnected behaviors occurring during the immediate postpartum period. Providing breastmilk as the first feed may help establish conditions that facilitate timely breastfeeding initiation, whereas the combination of these optimal breastfeeding practices has a potential to create a breastfeeding trajectory that supports sustained EBF [9], [74]. Importantly, this sequential pathway indicates that the effect of prenatal EBF intentions on EBF practice at 5 mo is not instantaneous but progressively depends on whether prenatal EBF intentions are successfully translated into specific postpartum behaviors. This finding may help explain why some mothers with strong prenatal EBF intentions do not ultimately achieve their breastfeeding goals when optimal breastfeeding practices are not established shortly after birth [72], [73], [75]. Our findings build on existing literature demonstrating that first feeding practices and early breastfeeding initiation function together as complementary mechanisms linking prenatal EBF intentions to EBF practice. From a public health perspective, these findings suggest that interventions targeting only 1 stage of the breastfeeding continuum may be insufficient [10], [74]. Rather, coordinated strategies that strengthen prenatal EBF intentions, promote provision of breastmilk as the first infant feed, and support breastfeeding initiation within the first hour after birth may be required to maximize EBF outcomes, particularly among mothers at increased risk of breastfeeding discontinuation [10], [34], [74].

### Study limitations

To our knowledge, this is the first study examining the mediating effects of first feeding practices and early breastfeeding initiation on the relationship between prenatal EBF intentions and EBF practice, providing novel insights into pathways through which prenatal EBF intentions influence later breastfeeding behavior. This contribution is strengthened by several methodological advantages, including the use of a large longitudinal cohort of WIC participants, a theory-driven sequential mediation analysis supported by sensitivity and subgroup analyses, and extensive adjustment for potential confounders. However, this study also had some limitations. As a secondary data analysis, the study was restricted to variables available in the ITFPS-2 study dataset. Consequently, it was not possible to account for some maternal and infant factors that could influence breastfeeding initiation and first feeding practices regardless of breastfeeding intention, such as infant oral anomalies and delayed lactogenesis. Also, the study population consisted of WIC participants and therefore may not be representative of higher-income households or the broader United States population. Further, the lack of a longitudinal survey weight variable at the 5-mo follow-up prevented the use of weighted analyses, which may affect the population-level generalizability [80]. Lastly, recall and social desirability bias may have affected some of the findings because most variables in the ITFPS-2 dataset were self-reported, potentially resulting in overreporting of recommended breastfeeding intentions and practices [55], [56]. Despite these limitations, our study provides valuable evidence on the sequential mediation pathways linking prenatal EBF intentions to EBF practice at 5 mo, offering potential targets for interventions aimed at improving EBF outcomes. Future studies could examine additional mediators and moderators not captured in the current study, including psychosocial factors such as social support, and maternal and child health factors that may influence the relationship between prenatal EBF intentions and EBF practice at 5 mo.

In conclusion, the association between prenatal EBF intention and EBF at 5 mo was partially mediated through sequential and individual pathways involving first feeding practices and early breastmilk initiation. Incorporating strategies that strengthen prenatal EBF intentions, promote early breastfeeding initiation, and discourage prelacteal feeding into WIC and other maternal–child nutrition support programs could improve EBF rates. These strategies may provide opportunities for enhanced prenatal breastfeeding counseling and targeted support during pregnancy and the immediate postpartum period, helping mothers establish and maintain the recommended optimal breastfeeding practices. If implemented successfully, such efforts could help achieve the United States national breastfeeding objectives, including those outlined in the Healthy People 2030 and the 2025–2030 DGA.

## Author contributions

The authors’ responsibilities were as follows – EJN, JJK: designed research objectives; EJN, JJK, T-LW: analyzed data; and all authors: made the final revision and took primary responsibility for the final content, wrote the paper, read and approved the final manuscript.

## Data availability

Deidentified data described and analyzed in this study are publicly available without any restriction through the USDA Food and Nutrition Service website at https://doi.org/10.15482/USDA.ADC/1528196.

## Declaration of generative AI and AI-assisted technologies in the writing process

The authors declare that no generative AI or AI-assisted technologies were used in the writing of this manuscript.

## Funding

This study was supported with research funds through the Mississippi Agricultural and Forestry Experimental Station (MAFES), Mississippi State University.

## Conflict of interest

The authors report no conflicts of interest. Content in this article is solely the responsibility of the authors and does not necessarily represent the official views of MAFES or Mississippi State University.
