# Supplementary material for: Sequential Mediation Effects of First Feeding Practices and Early Breastfeeding Initiation on the Relationship between Prenatal Exclusive Breastfeeding (EBF) Intentions and EBF at 5 Months: A Prospective Special Supplemental Nutrition Program for Women, Infants, and Children Program Infant and Toddler Feeding Practices Study-2
Source: Curr Dev Nutr. 2026 Oct 17;10(11):109557. doi: 10.1016/j.cdnut.2026.109557 (PMC13763428; doi:10.1016/j.cdnut.2026.109557)
Supplement: Supplementary file 1 [file mmc1.docx]

**Sequential Mediation Effects of First Feeding Practices and Early Breastfeeding Initiation on the Relationship Between Prenatal Exclusive Breastfeeding (EBF) Intentions and EBF at 5 Months: A Prospective WIC ITFPS 2 Study**

**Nsubuga**

**Online Supplementary Material**

**Supplemental Methods**

**The infant feeding intentions scale**

The scale scores range from 0 to 16, with 0 indicating no intention to breastfeed and 16 indicating a very strong intention to provide breast milk as the only source of infants’ food for the first 6 months (Table 7)(1). Respondents use a 5-point Likert scale (scored from 0 to 4) to answer five specific questions. The first 2 questions measure strength of intention to initiate breastfeeding i.e. I am planning to at least give breastfeeding a try and I am planning to only formula feed my baby (I will not breastfeed at all) respectively. The last 3 questions assess the strength of intention to provide the infant with human breast milk as the only infant food at 1, 3, and 6 months of age respectively (Table 7)(1). The total score is calculated by averaging the scores of the first 2-questions and summing that average with the scores of the last 3 questions (Table 7)(1).

| **Table 7: Infant Feeding Intention (IFI) Scale** (1) | | | | | | |
| --- | --- | --- | --- | --- | --- | --- |
| Instructions read to subject: I am going to read to you some statements about feeding your baby. Please choose the answer that most closely matches your opinion, considering both your feeding plans and the likelihood that you will carry out those plans. | | | | | | |
|  |  | Very much agree | Somewhat agree | Unsure | Somewhat disagree | Very much disagree |
| 1 | I am planning to only formula feed my baby (I will not breastfeed at all). | 0 | 1 | 2 | 3 | 4 |
| 2 | I am planning to at least give breastfeeding a try. | 4 | 3 | 2 | 1 | 0 |
| 3 | When my baby is 1 month old, I will be breastfeeding without using any formula or other milk. | 4 | 3 | 2 | 1 | 0 |
| 4 | When my baby is 3 months old, I will be breastfeeding without using any formula or other milk. | 4 | 3 | 2 | 1 | 0 |
| 5 | When my baby is 6 months old, I will be breastfeeding without using any formula or other milk. | 4 | 3 | 2 | 1 | 0 |
| *Numbers within grid represent the point value of each response. Total score = (mean of items 1 + 2) + (sum of items 3,4, and 5). This total score ranges from 0 (very strong intention to not breastfeed at all) to 16 (very strong intention to breastfeed exclusively throughout the first 6 months).* | | | | | | |

**Reference**

1.	Nommsen-Rivers LA, Dewey KG. Development and validation of the infant feeding intentions scale. Matern Child Health J. 2009;13(3):334-42.
